# Multiwalled Carbon Nanotubes-CeO_2_ Nanorods: A “Nanonetwork” Modified Electrode for Detecting Trace Rifampicin

**DOI:** 10.3390/nano10020391

**Published:** 2020-02-23

**Authors:** Na Zhang, Mariela Brites Helu, Keying Zhang, Xia Fang, Hu Yin, Jinmin Chen, Shangshang Ma, Aidong Fang, Cong Wang

**Affiliations:** 1 Anhui Key Laboratory of Spin Electron and Nanomaterials, School of Chemistry and Chemical Engineering, Suzhou University, Suzhou 234000, China; szxyzn@163.com (N.Z.); z2006590@163.com (X.F.); yh17605546701@163.com (H.Y.); jmchen@ahszu.edu.cn (J.C.); shangshangma@ahszu.edu.cn (S.M.); fad5006@163.com (A.F.); szxywangcong@163.com (C.W.); 2 Laboratoire de Chimie Physique et Microbiologie pour l’Environnement (LCPME), UMR 7564, CNRS—Université de Lorraine, 54600 Villers-les-Nancy, France; mariela.brites-helu@univ-lorraine.fr

**Keywords:** multiwalled carbon nanotubes, CeO_2_ nanorods, nanonetwork, rifampicin, modified electrode

## Abstract

Herein, a “nanonetwork” modified electrode was fabricated based on multiwalled carbon nanotubes and CeO_2_ nanorods. Scanning electron microscopy, X-ray powder diffraction and zeta potential were employed to characterize this electrode. Multiwalled carbon nanotubes negatively charged and CeO_2_ nanorods positively charged form “nanonetwork” via electrostatic interaction. The performance of the CeO_2_ nanorods-based electrode remarkably improved due to the introduction of multiwalled carbon nanotubes. The detection of rifampicin (RIF) was used as a model system to probe this novel electrode. The results showed a significant electrocatalytic activity for the redox reaction of RIF. Differential pulse voltammetry was used to detect rifampicin, the reduction peak current of rifampicin linear with the logarithm of their concentrations in the range of 1.0 × 10^−13^–1.0 × 10^−6^ mol/L, The linear equation is *i_p_* = 6.72 + 0. 46lg*c*, the detect limit is 3.4 × 10^−14^ mol/L (*S*/*N* = 3). Additionally, the modified electrode exhibits enduring stability, excellent reproducibility, and high selectivity. This strategy can be successfully used to detect trace rifampicin in samples with satisfactory results.

## 1. Introduction

Rifampicin (RIF) (3-{[(4-methyl-1-piperazinyl)imino]methyl}rifamycin), a bactericidal agent, is an important antibiotic drug that is often used to prevent the development of clinical tuberculosis [1]. Moreover, it has important applications in biological and pharmaceutical fields. Recent research demonstrates its efficiency in treating serious infections, such as human immunodeficiency virus (HIV) and cancer [2]. In the body, RIF is mainly metabolized in the liver through deacetylation and excreted in bile together with its metabolites [3]. RIF must be used with caution in the treatment of patients with liver diseases. Therefore, the development of more sensitive methods to detect RIF in pharmaceutical products and biological fluids is highly desired. 

Several methods have been reported for detecting RIF, such as high performance liquid chromatography [4], real-time polymerase chain reaction [5], chemiluminescence [6] and fluorescence spectroscopy [7,8,9]. Although these methods are generally quite sensitive and accurate, they are often costly, technically complex, time-consuming, and do not allow high throughput analysis. Recently, electrochemical methods, especially electrochemical RIF (rifampicin) sensors [10,11,12,13,14,15,16,17], have gained attention due to their outstanding merits, which includes the simplicity of the sample preparation, low cost of instrumentation, easy miniaturization, high sensitivity and selectivity. In particular, sensors based on metal oxide nanomaterials are well known for their excellent electrical, optical, thermal and catalytic properties, and large surface-to-volume ratio [18,19]. Cerium oxide (CeO_2_), an important rare earth material, is commonly seen in sensing due to its good biocompatibility, high stability and remarkable absorption capability [20]. In recent years, various morphologies of CeO_2_ including nanoparticles, nanorods, nanocubes, nanoshuttles, and nanoplates, have been synthesized seeking the enhancement of particular properties. For example, Kang et al. reported that CeO_2_ nanorods have a forceful adsorption capacity [21]. However, the low conductivity of CeO_2_ limits its further application in electrochemical sensing. Thus, it is highly desired to improve the conductivity of CeO_2_-based electrode for constructing electrochemical sensors.

In that sense, multiwalled carbon nanotubes (MWCNTs) are proposed for modifying and improving the performance of CeO_2_-based sensors. MWCNTs offer a large surface area, high mechanical strength, small diameter, good chemical and thermodynamic stability, and most importantly, excellent conductivity. MWCNTs can promote electron transport and increase the electrode surface area in electrochemical sensors as has been reported [22,23].

**Scheme 1:**
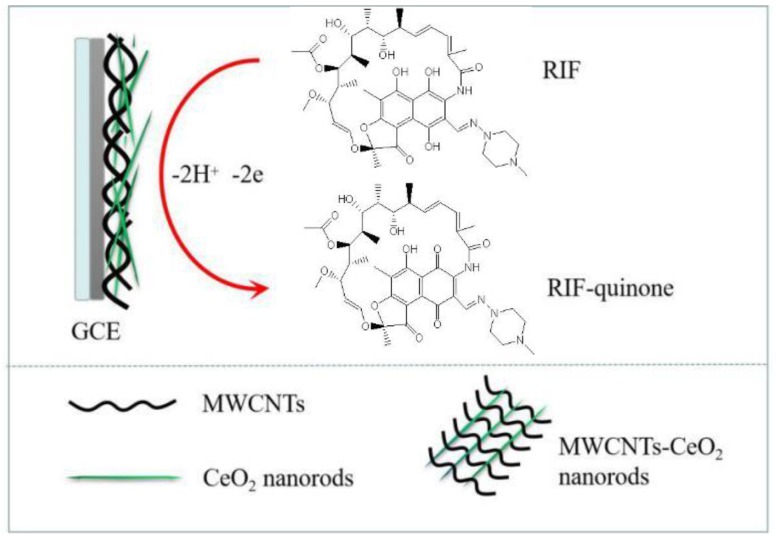
The scheme of the “nanonetwork” modified electrode and its electrocatalytic ability for the oxidation and reduction of RIF.

In this study, we combine the excellent properties of MWCNTs with the absorption capability of CeO_2_ nanorods to prepare an electrochemical RIF sensor with enhanced performance (Scheme 1). CeO_2_ nanorods with positive electrical charge are mixed with negatively charged MWCNTs to obtain a “nanonetwork” modified film. The results indicate that the “nanonetwork” increases the electrocatalytic activity for the oxidation and reduction of RIF. Moreover, the sensitivity is also increased, allowing to measure traces of RIF with satisfactory accuracy.

## 2. Materials and Methods

### 2.1. Reagents and Characterization

MWCNTs (purity >95%, diameter 20–30 nm, length 30 mm) were obtained from (Zhongke Nano New Material Co., Ltd., Shenzhen, China), CeCl_3_·7H_2_O was purchased from Sigma (Shanghai, China), RIF (USP grade), 0.1 M phosphate buffer solutions (PBS). Solution with different pH values were prepared by mixing the stock standard solution of Na_2_HPO_4_ and NaH_2_PO_4_ and pH was adjusted with H_3_PO_4_ or NaOH solution. All chemicals were analytical grade and were employed without further purification. Double distilled water was used in the all experiments.

The scanning electron microscope (SEM) images were obtained by Hitachi S-3000N (Japan Hitachi Co., Ltd., Tokyo, Japan); X-ray powder diffraction (XRD) measurements were performed on a Japan Shimadzu XRD-6000 diffractometer (Jiangsu, China) with Cu-Kα radiation (*λ* = 0.15418 nm) and a scanning rate of 0.05 deg. s^−1^; Zeta potentials were measured on a Nano-Z Zetasizer (Malvern Panalytical, Shanghai, China); Electrochemical measurements were carried out using CHI660A electrochemical workstation (Shanghai Chenhua Instrument Co., Ltd., Shanghai, China) with a three-electrode system (Nano-network” Modified Electrode as working electrode, platinum wire as the counter electrode and saturated calomel electrode (SCE) as reference electrode). All electrochemical measurements were carried out in a 10 mL electrochemical cell, where O_2_ was removed by bubbling high-purity N_2_ for 20 min. A continuous flow of N_2_ was maintained over the solution to avoid re-dissolution of O_2_ during measurements. All potentials given in this paper are referred to SCE. Each measurement was repeated three times to report statistical values.

### 2.2. Preparation of CeO_2_ Nano-Rods

CeO_2_ nanorods were synthesized as reported in previous work [24]. Briefly, 2.0 g CeCl_3_∙7H_2_O was dissolved in 10 mL water (solution a), 3.2 g NaOH was dissolved in 25 mL water (solution b), then both were mixed and stirred for 10 min. The solution was transferred to the reaction vessel and kept at 140 °C for 20 h. The product was rinsed with water and dried at room temperature for 20 h and calcined at 300 °C for 4 h to obtain CeO_2_ nanorods.

### 2.3. Preparation of MWCNTs-CeO_2_ Nano-Rods/GCE

A bare, glassy carbon electrode (GCE) was polished to a smooth, mirror-like finish with Al_2_O_3_ suspension, and cleaned by sonication in anhydrous EtOH and water for 1 min, each. Finally, the electrode was rinsed three times with water and dried at RT. The MWCNTs were used as received, MWCNTs/CeO_2_ nanorods suspensions were prepared with different ratios (1:4, 1:2, 1:1, 2:1, 3:1, weight ratios) by sonication during 30 min). The MWCNTs-CeO_2_ nanorods/GCE was prepared by drop-casting. Ten μL of suspension was dropped onto the GCE surface and dried at RT. 

## 3. Results and Discussion

### 3.1. SEM Characterization of MWCNTs-CeO_2_ Nano-Rods Composites

**Figure 1:**
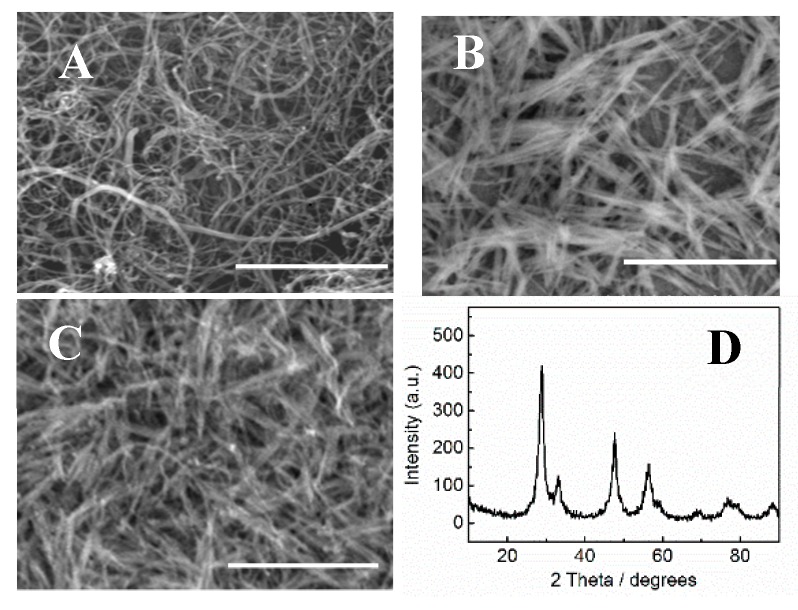
Scanning electron microscope (SEM) images of MWCNTs (**A**), CeO_2_ nanorods (**B**), and MWCNTs-CeO_2_ nanorods (**C**). Scale bar: 1.0 μm. The XRD of CeO_2_ nanorods (**D**).

Figure 1 shows the SEM images of CeO_2_ nanorods and MWCNTs-CeO_2_ nanorods “network” film. It can be seen from Figure 1A, MWCNTs were long and prone to entanglement. Figure 1B showed that the size of CeO_2_ nanorods were uniform, the zeta potential measurements showed that CeO_2_ nanorods were of the positive charge (≈ +28 mV). The XRD was used to characterize the CeO_2_ nanorods; these peaks matched the standard JCPDS No. 34-0394 (Figure 1D), indicating that CeO_2_ nanorods were successfully fabricated. Figure 1C showed the SEM image of MWCNTs-CeO_2_ nanorods film. Compared with the SEM of (A) and (B), the MWCNTs-CeO_2_ nanorods were dispersed and capable of forming a uniform “network” film.

### 3.2. Electrochemical Behaviors of RIF on Different Electrodes

**Figure 2:**
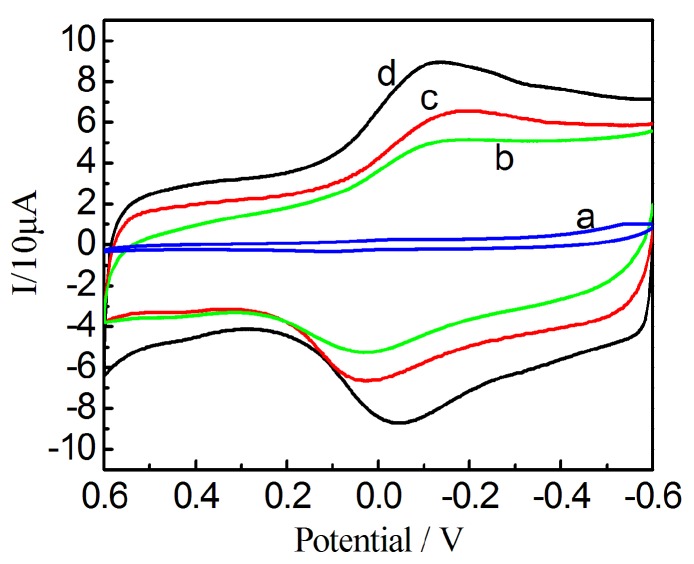
CVs of RIF on the different electrodes: Bare GCE (a); CeO_2_ nanorods/GCE (b); MWCNTs/GCE (c); MWCNTs-CeO_2_ nanorods/GCE (d) in 0.1 M phosphate buffffer solutions (PBS) (pH = 7.0). Scan rate: 0.10 V/s; the concentration of RIF: 1 × 10^−6^ mol/L.

Figure 2 shows several scans of cyclic voltammetry (CV) recorded on 10^−6^ mol/L RIF in 0.1 M PBS (pH = 7.0) at different electrodes material: bare GCE (a), CeO_2_ nanorods/GCE (b), MWCNTs/GCE (c), and MWCNTs-CeO_2_ nanorods/GCE (d). Bare GCE shows virtually no redox activity for RIF as the CV scan does not show a peak pair in curve 2-a. By modifying the electrode with MWCNTs and CeO_2_ nanorods, the peaks for the reversible oxidation and reduction of RIF appears, which is evidence of the enhancement on the activity. For CeO_2_ nanorods/GCE and MWCNTs/GCE, the oxidation peak appears ca. −0.1 V vs. SCE followed by a reduction peak ca. 0.05 V vs. SCE in the reverse scan. The peak currents are further increased on MWCNTs-CeO_2_ nanorods /GCE (curve 2-d). In this case, it is also seen a shift in the peak potential to more negative values. This may be due to: (1) MWCNTs and CeO_2_ nanorods have large area which can increase the effective surface area of the electrode, which yield to more activation sites for the reaction of RIF, (2) MWCNTs with good electrical conductivity can improve the electron transfer ability of the electrode; (3) CeO_2_ nanorods can increase the adsorption amount of RIF on the modified electrode surface due to its ability to bind to oxygen-rich groups, which could improve the detection signal as well.

### 3.3. Optimization of the Ratio of MWCNTs/CeO_2_ Nano-Rods

**Figure 3:**
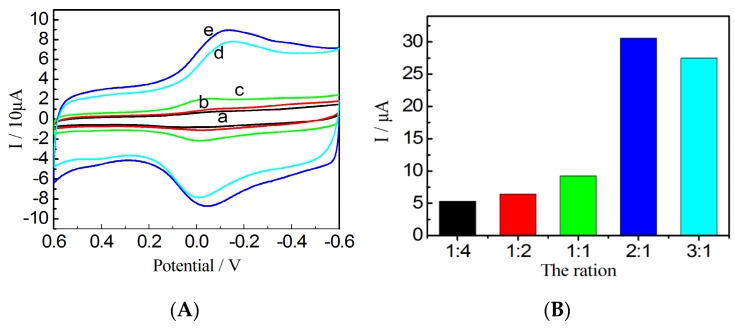
(**A**) CVs of the electrode modified with different ratio of MWCNTs/CeO_2_ nanorods: 1:4 (a); 1:2 (b); 1:1 (c); 2:1 (e); 3:1 (d). (**B**) The relationship between the reduction peak current and the ratio of MWCNTs/CeO_2_ nanorods.

The ratio of MWCNTs/CeO_2_ nanorods is a key factor that affects the electrocatalytic activity of the modified electrode. Figure 3 shows the effect of the ratio on the current peak magnitude. The composite with a weight ratio 2:1 exhibits the major enhancement on the activity. Therefore, this composition was selected to further investigate the performance of MWCNTs-CeO_2_ nanorods/GCE.

### 3.4. Effect of pH

**Figure 4:**
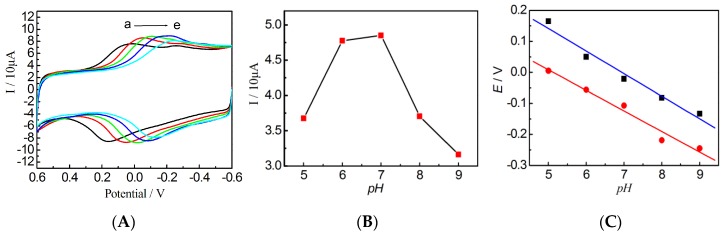
(**A**) CVs of RIF at the MWCNTs-CeO_2_ nanorods/GCE in 0.1 M PBS with different pH (a—5.0, b—6.0, c—7.0, d—8.0, e—9.0); (**B**) The relationship between the reduction peak current and pH; (**C**) The relationship between the peak potential and pH (The reduction peak potential (blue) and The oxidation peak potential (red)). Scan rate: 0.10 V/s.

**Scheme 2:**
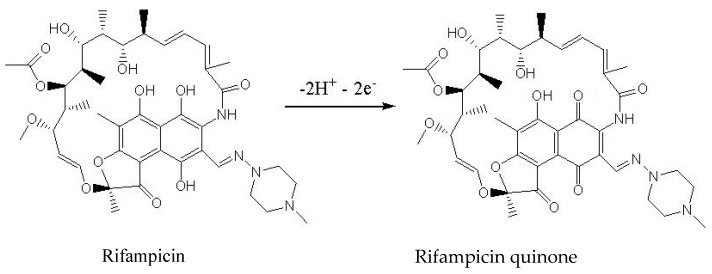
The electrochemical reaction mechanism of RIF at the surface of MWCNTs-CeO_2_ nanorods/GCE.

The effect of the pH of the solution on the electrochemical response of RIF was investigated by CV. Figure 4 shows scans of MWCNTs-CeO_2_ nanorods/GCE-RIF carried out under different pH conditions within the range of 5.0–9.0. Both, the current and the potential peaks are affected by the pH. As shown in Figure 4B the reduction peak current of RIF reaches is maximum value in neutral solutions (pH = 7). Thus, PBS buffer has to be added to maintain the pH at 7 during the measurements. In addition, the anodic and cathodic peaks shift to more negative values when the pH increases. This has a clear lineal relationship, as shown in Figure 4C. The linear regression equation is *E*_pa_ = 0.51 − 0.072pH (*R*^2^ = 0.9862) for the anodic process and *E*_pc_ = 0.34 − 0.066pH (*R*^2^ = 0.9859) for the cathodic process. These results show the the participation of protons in the electrochemical reaction mechanism. The path for the reaction is shown in Scheme 1. Similar results and hypothesis were reported in previous work [10] (Scheme 2). 

### 3.5. Effect of Scan Rate

**Figure 5:**
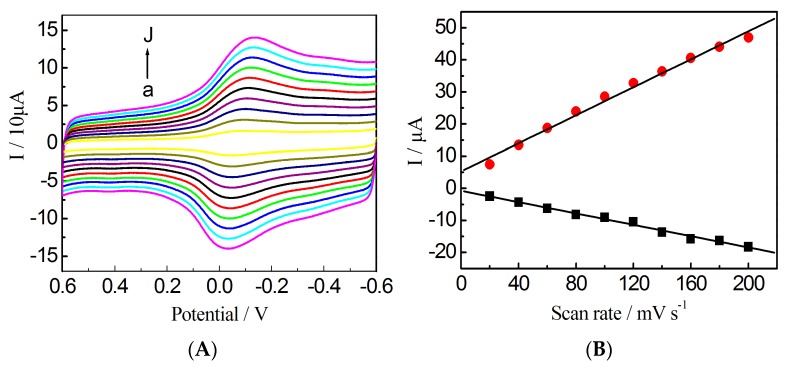
(**A**) CVs of RIF at the MWCNTs-CeO_2_ nanorods/GCE at the different scan rates in 0.1 mol/L PBS (a—0.02 V/s, b—0.04 V/s, c—0.06 V/s, d—0.08 V/s, e—0.10 V/s, f—0.12 V/s, g—0.14 V/s, h—0.16 V/s, i—0.18 V/s, j—0.20 V/s). (**B**) The relationship between the redox peak currents and scan rate.

The effect of scan rate (*v*) on the electrochemical behaviors of RIF on the MWCNTs-CeO_2_ nanorods/GCE was investigated. Figure 5A shows the CV responses of RIF at MWCNTs-CeO_2_ nanorods/GCE within *v* = 0.02–0.20 V/s range. It is clearly seen that the cathodic and anodic current peaks are increased as the scan rate does. In both cases, a linear behavior is exhibited as is shown in Figure 5B. The linear equations are *i*_pa_ (μA) = −0.76 − 0.09*v* (*R*^2^ = 0.9951) and *i*_pc_ (μA) = 5.25 + 0.22*v* (*R*^2^ = 0.9953), for the anodic and cathodic peaks, respectively. These results indicate that the reaction process is controlled by adsorption.

### 3.6. Standard Curve and the Detection Limit

**Figure 6:**
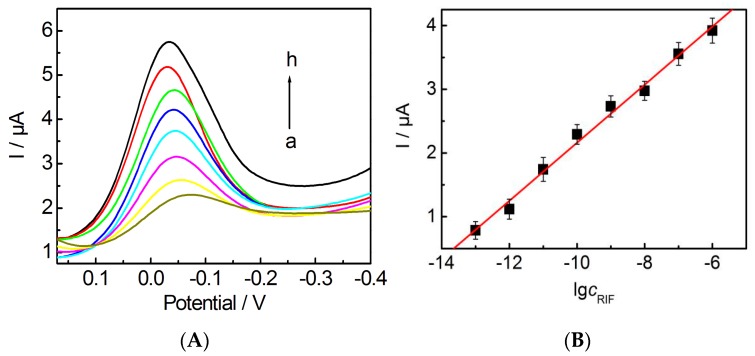
(**A**) DPVs of the different concentrations of RIF at MWCNTs-CeO_2_ nanorods/GCE in 0.1 mol/L PBS (pH = 7.0); (**B**) The reduction peak current vs. the concentration of RIF. a—1.0 × 10^−13^, b—1.0 × 10^−12^, c—1.0 × 10^−11^, d—1.0 × 10^−10^, e—1.0 × 10^−9^, f—1.0 × 10^−8^, g—1.0 × 10^−7^, h—1.0 × 10^−6^ mol/L.

Under the optimal experimental conditions, the calibration curve and detection limit were carried out by differential pulse voltammetry (DPV) in 0.1 mol/L PBS. It can be seen from Figure 6A that RIF oxidation peak increases as the concentration is increased, showing a linear relation with the logarithm of RIF concentration in the range from 1.0 × 10^−13^ to 1.0 × 10^−6^ mol/L. The obtained linear equation is *i_p_* = 6.72 + 0.46lg*c* with the linear coefficient (*R*^2^) equals to 0.9964. Moreover, the minimum concentration that can be accurately detected (namely, detection limit) is 3.4 × 10^−14^ mol/L (with a signal/ nose ratio *S*/*N* = 3). The obtained linear range and detection limit are compared with data from previous RIF sensors in Table 1. In comparison, the method proposed here has a much better performance with a larger lineal range of detection. More importantly, the limit detection has been decreased thousands of times, positioning the MWCNTs-CeO_2_ nanorods/GCE sensor as a promising tool in assay RIF in biological and pharmaceutical samples.

### 3.7. Reproducibility and Stability of Modified Electrode

The reproducibility and stability of modified electrode was studied. Three MWCNTs-CeO_2_ nanorods/GCE were independently prepared and employed to detect the same reference solution of RIF with a concentration of 1.0 × 10^−^^9^ mol/L. The relative standard deviation found was equal to 3.7%, which indicates that the modified electrode has an excellent reproducibility. In addition, the modified electrode was kept in 0.1 mol/L PBS for 4 consecutive days at room temperature and used for measuring the reference solution, once a day. The electrochemical signal was quite stable during this period with values that represent the 97%, 93%, 91%, and 90% of the original measured value.

### 3.8. Interference Studies and Samples Analysis

Several concomitants were added to the RIF solution to study their interference during the detection of RIF. Uric acid, *L*-threonine, and glucose were selected as potential interference to evaluate the selectivity of the modified electrode for detecting RIF in pharmaceutical formulations and the biological fluids. The test results indicate that concentration of uric acid, *L*-threonine and glucose up to 600-fold the RIF concentration do not have an effect on the DPV detection signal of RIF. Therefore, the MWCNTs-CeO_2_ nanorods/GCE sensor can be used to determine RIF in human serum. Analyisis of complex samples by the standard addition method were carried out. The recovery data is shown in Table 2. The values are in the range of 94.6–102.2%, indicating the constructed method can be applied to detect RIF in the complex samples.

## 4. Conclusions

Herein, a “nanonetwork” modified electrode was fabricated based on MWCNTs and CeO_2_ nanorods, and employed to detect RIF. The electrochemical behavior and reaction mechanism of RIF at this modified electrode surface was studied. The experiment results imply that the proposed electrode have obvious electrocatalytic ability for the redox of RIF. Additionally, the proposed method has high sensitivity and selectivity, and has been successfully applied for detecting RIF in complex samples, thus demonstrating its potential application in the assay of RIF in biological and pharmaceutical samples.

## Figures and Tables

**Table 1:** Comparison of the linear ranges and detection limits of previous RIF sensors.

Modified Electrode	Linear Range (mol/L)	Detection Limit (mol/L)	Ref.
Gold nanoparticles/poly-melamine nanocomposite	8.0 × 10^−^^8^–1.5 × 10^−^^5^	3.0 × 10^−^^8^	[12]
Graphene nanoplatelets	1.0 × 10^−^^9^–1.0 × 10^−^^4^	5.0 × 10^−^^10^	[13]
Copper metal organic framework/mesoporous carbon	8.0 × 10^−^^8^–8.5 × 10^−^^5^	2.8 × 10^−^^10^	[15]
MWCNTs/meso-tetrakis(4-hydroxyphenyl)porphyrinato cobalt(II)	1.0 × 10^−^^8^–5.0 × 10^−^^3^	8.0 × 10^−^^9^	[17]
MWCNTs-CeO_2_ nanorods/GCE	1.0 × 10^−13^–1.0 × 10^−6^	3.4 × 10^−14^	This work

**Table 2:** Determination of RIF in serum samples (*n* = 3).

Sample	Added (pmol L^−1^)	Found (pmol L^−1^)	Recovery (%)	R.S.D. (%)
1	1.0	0.97	97.0	1.97
2	10.0	9.46	94.6	1.87
3	50.0	51.1	102.2	2.02
